# Investigating the overlap of machine learning algorithms in the final results of RNA-seq analysis on gene expression estimation

**DOI:** 10.1007/s13755-023-00265-4

**Published:** 2024-02-29

**Authors:** Kalliopi-Maria Stathopoulou, Spiros Georgakopoulos, Sotiris Tasoulis, Vassilis P. Plagianakos

**Affiliations:** 1 Department of Computer Science and Biomedical Informatics, University of Thessaly, Papasiopoulou 2-4, 35100 Lamia, Greece; 2 Department of Mathematics, University of Thessaly, Volos, Greece

**Keywords:** Differentially expressed genes, Gene expression, Machine learning, Supervised, Unsupervised, RNA-seq analysis, NGS data, Bioconductor

## Abstract

Advances in computer science in combination with the next-generation sequencing have introduced a new era in biology, enabling advanced state-of-the-art analysis of complex biological data. Bioinformatics is evolving as a union field between computer Science and biology, enabling the representation, storage, management, analysis and exploration of many types of data with a plethora of machine learning algorithms and computing tools. In this study, we used machine learning algorithms to detect differentially expressed genes between different types of cancer and showing the existence overlap to final results from RNA-sequencing analysis. The datasets were obtained from the National Center for Biotechnology Information resource. Specifically, dataset GSE68086 which corresponds to PMID:200,068,086. This dataset consists of 171 blood platelet samples collected from patients with six different tumors and healthy individuals. All steps for RNA-sequencing analysis (preprocessing, read alignment, transcriptome reconstruction, expression quantification and differential expression analysis) were followed. Machine Learning- based Random Forest and Gradient Boosting algorithms were applied to predict significant genes. The Rstudio statistical tool was used for the analysis.

## Introduction

RNA-sequencing (RNA-seq) is a powerful technique for characterizing and quantifying the transcriptome and accelerates the development of bioinformatics software. Due to the low cost of next-generation sequencing technologies and remarkable power and accuracy, RNA sequencing has become the most popular method for analyzing differentially expressed genes [1]. The workflow of the next generation of RNA sequencing [2] data includes the preprocessing and the downstream analysis. Machine Learning is a multidisciplinary field that uses computer science, computational statistics, and information theory to build algorithms that can learn from existing datasets and make predictions for new datasets [3]. Machine Learning is a key tool for biological studies, including the study of cancer as well as the discovery of genes.

This article analyzes differential gene expression in a large RNA-seq dataset using samples with different types of cancer and normal conditions and examines the discovery of important genes identified between the use of classical RNA-seq analysis and machine learning algorithms. The scientific question we analyze in the present study is the reliability and overlap of machine learning algorithms in the final outcome of an RNA-seq analysis. More specifically, in the aforementioned experiment (cancer vs normal) 4,559 genes were identified by RNA-seq analysis and in combination with 2 different classification algorithms, we identified common important differentially expressed genes. We found that there is reproducibility and overlap between the two methods for finding the most significant differentially expressed genes which play an important role in the development of cancerous tumors that we analyze. Also, we found that the random forest [4] and gradient boosting [5] models are powerful for predicting differentially expressed genes. Raw data from cancer patients were extracted from the NCBI-GEO database [6]. The analysis was performed with the use of the R programming language and RStudio user interface to execute the code and visualize the results. R is one of the well-known programming languages and is an open-source software developed by the scientific community for calculating, analyzing and visualizing big data in any field, including biomedical research for bioinformatics applications. R with the help of Bioconductor in RStudio provides many packages that support high-performance sequence data analysis, including RNA sequencing (RNA-seq) [7].

Overall, our study shows that combining machine learning with RNA sequencing has significantly improved the recognition of the most important differentially expressed genes.

## Materials and methods

For the RNA-seq analysis, the workflow we have taken into account includes the following steps:

1. Data acquisition (obtained raw RNA-seq data).
2. Data Quality Control (First QC of RNA-seq analysis). We assessed the quality of our raw data using tools like FastQC to check for sequencing errors, adapter contamination, and other issues.
3. Preprocessing (Trim adapters and low-quality bases from the raw reads using tool like Trimmomatic).
4. Read Alignment (Second QC of RNA-seq analysis). We aligned the cleaned and trimmed sequencing reads to a reference genome.
5. Quantification (We estimated gene and transcript expression levels using tools like featureCounts and Salmon. This step produces count tables that represent how many reads map to each gene or transcript).
6. Normalization and QC (Third QC of RNA-seq analysis). We normalized expression counts to account for variations in library size and composition. Common normalization methods include TPM (Transcripts Per Million) or FPKM (Fragments Per Kilobase Million).
7. Differential Expression Analysis (We identified genes that are differentially expressed between different experimental conditions using DESeq2 software. And we performed statistical tests to assess significance, calculate fold changes, and generate lists of differentially expressed genes).
8. Functional Enrichment Analysis (We interpreted the biological significance of differentially expressed genes by performing functional enrichment analysis using tools like Gene Ontology (GO) analysis, pathway analysis (KEGG), or gene set enrichment analysis (GSEA).
9. Biological Interpretation (We interpreted the results in the context of our biological question. Investigated the biological functions and pathways associated with differentially expressed genes).

The methods used to analyze RNA sequencing include obtaining raw RNA-seq data in fastq format from the GEO database (GEO Accession Number GSE68086). From the 171 total samples, 35 regard breast cancer, 11 liver cancer, 30 colorectal cancer, 13 glioblastoma, 33 lung cancer, 25 pancreatic cancer and 24 healthy individuals. Quality control was performed in RStudio for each sample separately with the FastQC tool from the Babraham institute bioinformatics group, which is used to evaluate the quality of the sequence data [8]. Following quality control, Trimmomatic was used for filtering samples [9]. The reads were mapped with Rsubread 2.0.1 [10] to RStudio, using the hg38 human genome and the GTF annotation file. Quantification of gene expression was performed using Salmon [11], which correlated sequence readings directly with transcripts. The differential expression of the genes was completed with DESeq2 [12] in RStudio and the genes were annotated with Bioconductor annotation packages, which help to map different identification systems (ID) between them. The AnnotationDbi and org.Hs.eg.db libraries were used to annotate on the differentially expressed genes. Finally, using gProfileR [13] we did the functional enrichment analysis on the Gene Ontology (GO) terms. We also identified the most important genes expressed in GO terms through the gage and pathview packages. We also did pathway analysis based on the KEGG database [14].

The classification methods used for the Machine Learning analysis include the caret package in RStudio [15], which was used to train and evaluate the algorithms.

The workflow for Random Forest and Gradient Boosting in RNA-seq analysis that we used to detect differentially expressed genes in various types of cancer is:

1. Data Preprocessing (Obtained RNA-seq data and performed quality control and data preprocessing, including read alignment, transcript quantification and normalization).
2. Feature Selection (Genes are quantified from the aligned reads to create a count matrix. We used statistical methods and machine learning -based feature selection techniques to choose the most important genes).
3. Labeling (Assigned labels to the samples based on experimental conditions).
4. Data Splitting (Split the dataset into training and testing sets. Training set is used to train the models and the testing set is used for model evaluation).
5. Model Selection (Random Forest and Gradient Boosting models are chosen as potential machine learning algorithms for the analysis).
6. Hyperparameter Tuning (We used techniques like grid search and random search to optimize the parameters of the chosen models. Optimize parameters such as the number of trees, max depth and learning rate for Gradient Boosting).
7. Model Training (The models trained on the training dataset using the optimized hyperparameters).
8. Model Evaluation (The trained models evaluated on the testing / validation dataset using appropriate metrics like accuracy, recall, F1-score, ROC curves, precision, etc.).
9. Feature Importance Analysis (Extracted feature importance scores generated by the models to identify the most relevant genes in our RNA-seq data).
10. Overlap with RNA-seq Analysis (Compared the genes identified by our machine learning models with the results of our RNA-seq analysis to find the overlap).
11. Biological Interpretation (Interpreted the results in the context of cancer biology to understand the functional significance of the differentially expressed genes. Also, identified potential pathways).

## Results

The Fastqc tool was used for the quality control step. 76 samples passed the quality score which was over 30, while 95 samples did not meet the desired quality grade. Trimmomatic was used to remove areas (trimming) of the reads, whereas the Rsubread package was used to map the reads to the reference genome. BAM files were created and checked for alignment quality and showed a minimum mapping quality of 34, which is sufficient. Quantification of gene expression was performed using Salmon. Training data included the quant.sf file for each of the 129 samples from the Salmon output. We imported quant.sf files using tximport, scaling transcripts-per-million (TPM) using the average transcript length across samples and the library size (length-Scaled TPM), followed by the log_2_ transformation. In the remaining 42 of the 171 samples there were no quantification results because they showed an error in reading fastq. This is the smallest return of the code from both the right and the left of the reading was (− 2), indicating that the files are not valid and therefore could not be quantified. The percentage of aligned reads from all samples is 71 to 84%. The total number of reads of the samples corresponding to 561,501,702 readings was obtained using DESeq2. The unexpressed genes were filtered, and reasonable values were obtained, that show how many samples each gene are expressed. The resulting table refers to 129 samples with 35,135 genes. The data was afterwards normalized and filtered, while the library size was reduced and the dependence of the variance on the mean was removed. Thus, out of the 35,135 genes, 10,796 that were expressed in all 129 samples were kept for further analysis. For the result of 10,796 genes, automatic filtering was performed based on the average of the normalized measurements for each gene. DESeq2 and the Benjamini–Hochberg (BH) false discovery rate (fdr) for multiple hypothesis testing correction were used to calculate the fdr adjusted p value for each gene. The study is limited to 10,796 genes that were expressed in all samples, because the main goal of the study was to find genes that are important and expressed in all types of cancer that we examined.

**Fig. 1:**
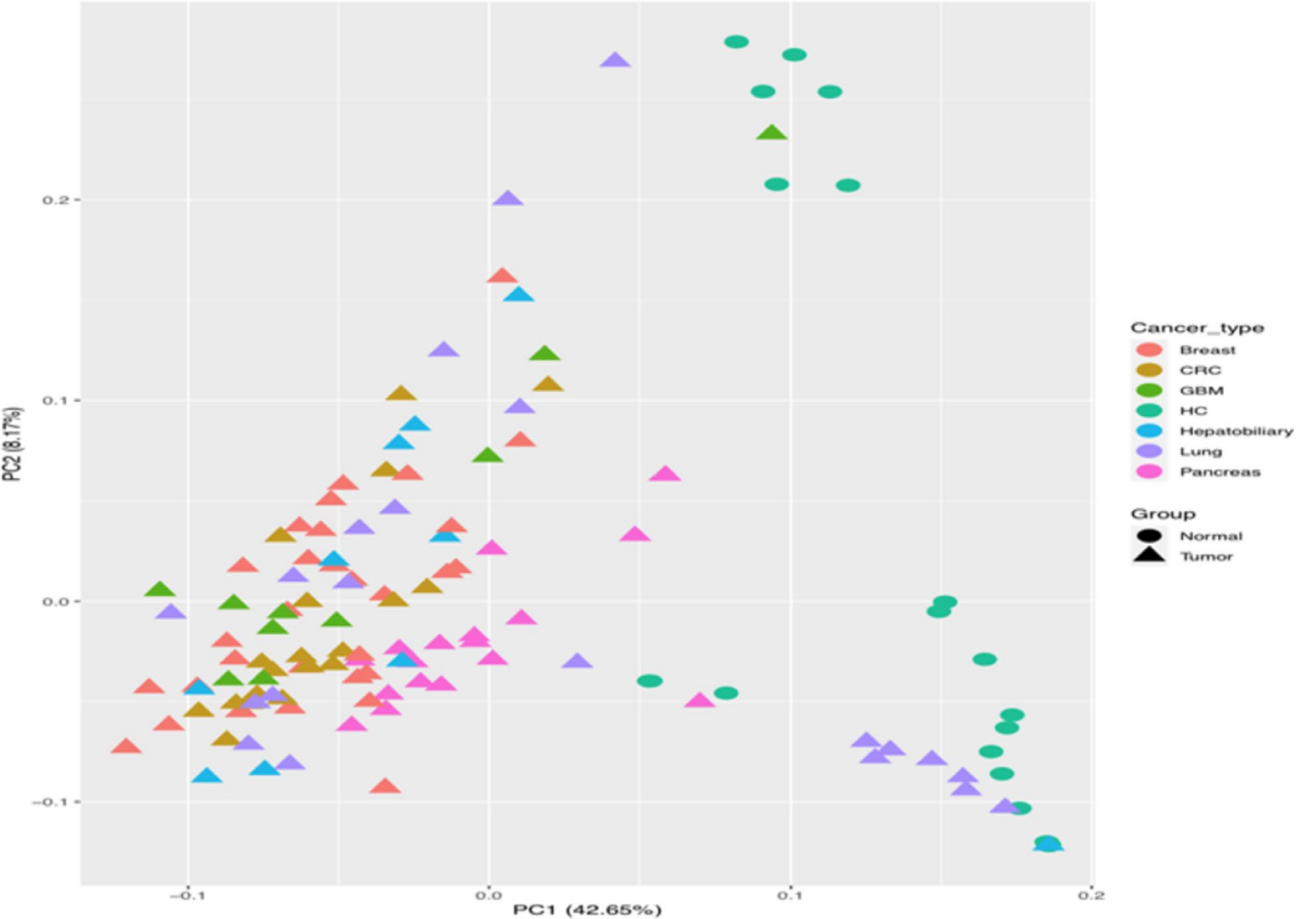
Principal component analysis plot. From the above plot we see that the differences between the two conditions (Tumor and Normal) are significant. The samples concerning healthy people have short distances from each other in relation to other types of cancer. Also, some distances are observed between lung cancer samples compared to other types of cancer

For the third quality control and evaluation of the whole experiment, the Principal Component Analysis (PCA) plot, as shown in Fig. 1, for the samples’ distance was used. The PCA plot was performed with DESeq2 which offers the variance stabilization transform (VST) for negative binomial data. This means that the differences between the normal samples and the tumor will contribute to the expected mean variation of the experiment. The graph also shows the samples in the 2D plane extending from its first two main components, where the first dimension concerns the separation of cancer types in the samples and the second dimension concerns the separation of samples into tumor data sets from normal data sets.

**Fig. 2:**
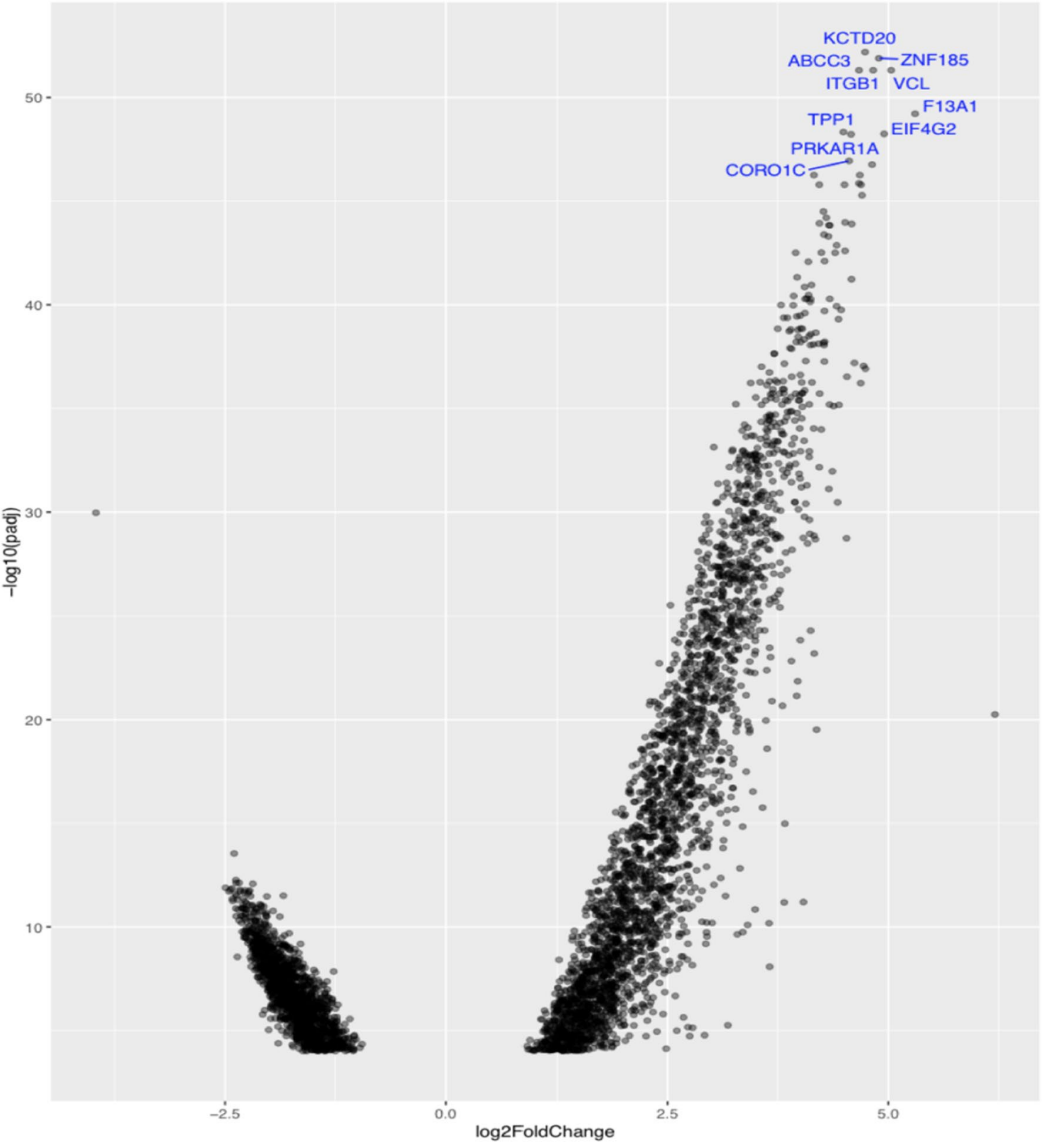
The Volcano plot with the 4,559 most differentially expressed genes. The most upregulated genes are to the right and the most downregulated genes are to the left. The highest upregulated genes are at the top. The 10 of the most important differentially expressed genes are *KCTD20*, *ZNF185*, *VCL*, *ITGB1*, *F13A1*, *TPP1*, *EIF4G2*, *PRKAR1A*, *ABCC3* and *CORO1C*

DESeq2 was used for differential gene expression in R. The dispersion estimation, Wald statistic was performed, where the negative binomial model for each gene was placed and the nbinomWaldTest was used to control differential expression. 10,796 genes were found with a significant change in gene expression between samples and after filtering the p value with p-value < 0.0001, recovering the normalized measurements and comparing tumor versus normal samples, the 4,559 genes were found to be the most important differentially expressed. Ensembl transcript names were converted into gene symbols using the AnnotationDbi package. To visualize the most important differentially expressed genes, the Volcano Plot was created, which shows the relationship of expression change between the two conditions. The Volcano plot, as shown in Fig. 2, is a type of scatter plot that shows statistical significance (p value) versus fold change. It allows fast visual recognition of genes with changes that are statistically significant.

Functional enrichment analysis was performed with the gProfileR package and adjusted value p < 0.0001. Significantly enriched GO terms were identified, of which 135 were overrepresented and classified into 94 Biological Process (BP), 11 Molecular Function (MF) and 30 Cellular Components (CC), respectively. The most important term GO in frequency and uniqueness was found in the term GO: 0005515, which belongs to the group MF called protein binding, with a frequency of 76.4%. Among all GO terms, the most important upregulated genes are *KCTD20*, *ZNF185*, *VCL*, *ITGB1*, *F13A1*, *TPP1*, *EIF4G2*, *PRKAR1A*, *CORO1C* and the most important downregulated genes are *GPNMB*, *ZNF835*, *MARN22*. The enrichment analysis of the gene sets with the gage package was then performed. A total of 156 KEGG pathways were identified with an adjusted value of p < 0.0001, of which 7 were upregulated and 149 were downregulated. The differentially expressed genes were gathered in the following pathways, hsa00190-oxidative phosphorylation, hsa04145-phagosome, hsa04810-regulation of actin cytoskeleton, hsa04510-focal adhesion, hsa04670- leukocyte interstitial migration and hsa004144-endocytosis. Among all pathways, the most important upregulated genes are *ITGB1*, *VCL*, *CORO1C*, *ABCC3*, *F2R*, *ACTN1*, *CDC42*, *GRB2*, *EHD3*, *NDUFA4* and downregulated are *NDUFV3*, *CORO2A*, *TLR6*, *SL LDLR*. GO enrichment analysis revealed that the predicted regulatory gene group was enriched with genes involved in differential expression analysis.

Subsequently, two supervised learning algorithms were tested. The choice of these two algorithms was made after analyzing many algorithms. It is common to explore multiple algorithms and techniques during RNA-seq analysis to determine which one(s) provide the best results for our specific research question and data set. Furthermore, feature engineering, data preprocessing, and cross-validation play critical roles in the success of machine learning in RNA-seq analysis. Random Forest and Gradient Boosting algorithms in addition to the results were found to be suitable and fit our research objective because we prioritized robustness, feature importance analysis—providing feature importance scores, and high predictive accuracy.

We used Random Forest because it is a powerful, easy-to-implement model that handles high-dimensional data and provides feature importance scores for gene selection. It is robust to outliers and noisy data. This is beneficial in RNA-seq analysis, where gene expression data can have variations and technical noise. It can be used for binary or multiclass classification of genes based on their expression patterns in different cancer types.

We used the Gradient Boosting algorithm because it offers high prediction accuracy which is critical in RNA-seq analysis and can be used for accurate gene classification and feature selection, helping us identify differentially expressed genes. It can handle noisy data by iteratively improving predictions and reducing the impact of outliers. And if the RNA-seq dataset has a limited number of samples, Gradient Boosting can perform well due to its iterative nature and focus on correcting misclassifications.

Before starting training, exploratory data analysis was done to see how the variables and samples were related to each other. The first thing we did is data normalization and transformation. We took care of data scaling issues that might come from how the experiments were run and potential problems that might arise during data collection. The next step was to transfer our data. We then filtered the predictor variables and selected arbitrary cutoffs for variability. The expression values of the initial 35,135 genes were used. To execute the code, the caret::preProcess function was used to filter the predictor variables, the 1,000 best predictors were selected, that is the gene expression values, and then we filtered the highly related prediction variables, creating a filter for the subtraction of related variables. If two variables are sufficiently correlated, only one of them is removed. The classification problem used was binary and involved tumor samples versus normal samples. Of the 129 samples, 108 correspond to cancer samples (30 breast, 10 liver, 19 colorectal, 21 lung, 18 pancreatic, 10 glioblastoma) and 21 are normal samples. The training and testing of the data was done with the method caret::createDataPartition, where the parameter p = 0.7 was set, meaning that training to test ratio is 70:30. This corresponds to 91 samples for the training set and 38 samples for the test set.

To configure the Random Forest and Gradient Boosting algorithms we included the setting of various hyperparameters such as:

### RandomForest

1. n_estimators: This parameter defines the number of decision trees in the forest. We used “ranger” method and the argument tuneGrid in the “train” function, which specifies a grid of parameters.
2. mtry was set to 100, which is the number of variables randomly selected at each split in each tree. This value is part of the hyperparameter tuning process.
3. Criterion: Random Forest can use different criteria for splitting nodes in the decision trees. Splitrlule was set to “gini”, indicating that the Gini impurity criterion was used for splitting nodes in the decision trees.
4. min_samples_leaf: Sets the minimum number of samples required to be at a leaf node. In our code min.node.size was set to 1.
5. bootstrap: A Boolean parameter indicating whether to use bootstrapping when building trees. Bootstrapping introduces randomness into the model, which can reduce overfitting. We did not explicitly set bootstrap, the default value < TRUE > was used.
6. random_state: Controls the random seed for reproducibility.

We set the random seed to 17 using set.seed (17).

### Gradient boosting

1. n_estimators: This parameter defines the number of boosting stages (trees) to use. In our code, we set nrounds = 200 in the tuneGrid, which corresponds to the number of trees in the ensemble.
2. Learning_rate: Determines the step size at each iteration while moving toward a minimum of a loss function. The code specifies a range of values for the learning rate (0.05, 0.1, 0.3), indicating that it is likely being tuned during the cross-validation process.
3. max_depth: The maximum depth of individual trees. The code specifies a range of values for the maximum depth of trees (max_depth = 4).
4. We set gamma = 0, which is a regularization parameter that controls the complexity of individual trees.
5. subsample: The code specifies a value of 0.5 for subsampling, indicating that a fraction of samples is used for fitting the trees.
6. We set the minimum sum of instance weigh (hessian) needed in a child. min_child_weight = 1. It’s a regularization parameter.
7. random_state: Controls the random seed for reproducibility, just like in Random Forest. We set the random seed to 17 using set.seed (17).

Based on our data, we modeled these hyperparameters, using techniques such as grid search or random search, and tested various combinations of hyperparameters to find the best set for our data. Also, variable importance was calculated and plotted.

More specifically, we tested the Random Forest algorithm with 100% success rates for the training set and 84.21% for the test set and the Gradient Boosting algorithm with 98.9% success rates for the training set and 86.8% for the test set.

**Fig. 3:**
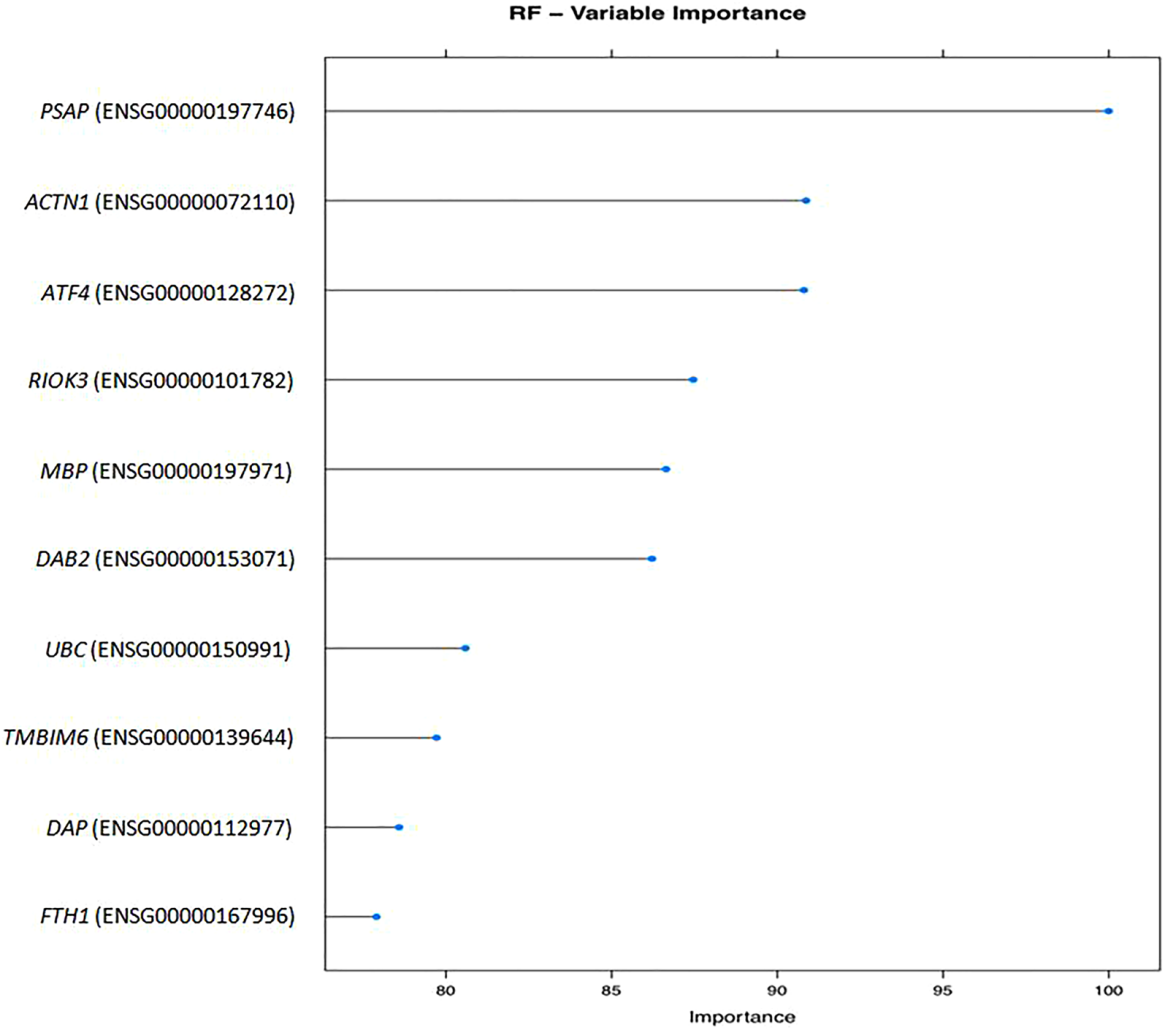
Random forest algorithm plot. The above plot indicates the overlap of the Random Forest algorithm with the most important genes (identifiers) from the results of differential expression

**Fig. 4:**
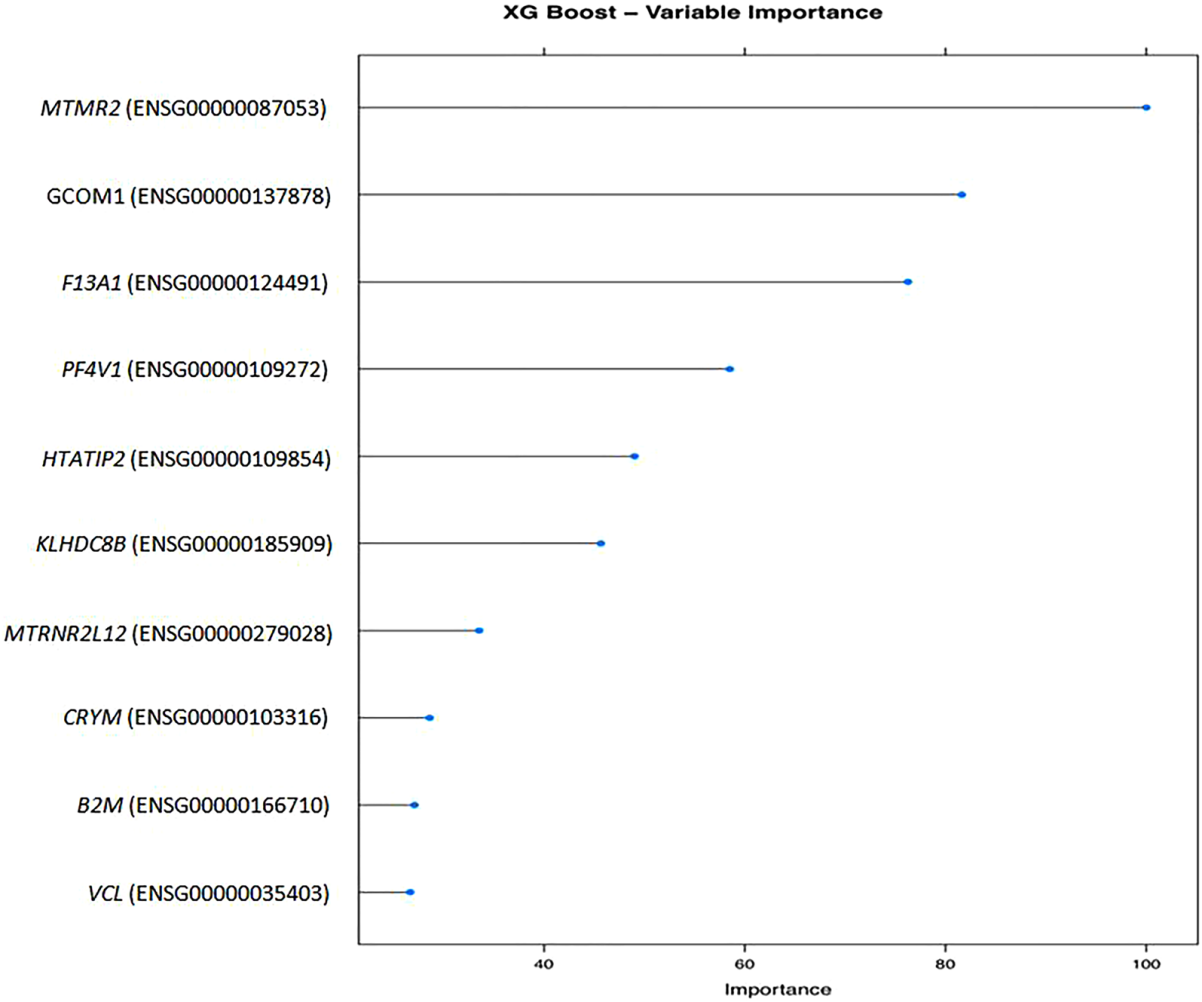
Extreme Gradient Boosting algorithm plot. The above plot indicates the overlap of the Gradient Boosting algorithm with the most important genes (identifiers) from the results of differential expression

Experimental results indicate that both classifiers had good results. But most importantly, the variables were checked with the Random Forest algorithm (Fig. 3) and Gradient Boosting algorithm (Fig. 4) and the result was that there is an overlap with the most important genes from the results of differential expression and functional enrichment of the genes (GO terms and KEGG pathways). The genes commonly found were *VCL*, *F13A1* and *ACTN1*.

## Discussion

Several bioinformatics studies have made RNA-seq analysis [16] and comparisons with RNA-seq and machine learning algorithms such as the study which addresses practically machine learning based approach for gene expression analysis using RNA-seq data for cancer research and compare it with a classical gene expression analysis approach [16, 17]. Many researchers have used algorithms for the detection of important differentially expressed genes [18, 19], for the identification and recovery of reference transcripts with high precision, resulting in high—quality normalization [20], for the detection of gene signatures which are important for better research and clinical treatment [21], for sequence analysis about the sequence alignment and the prediction of RNA structure [22] and many other analyses.

Important papers that we considered in our study are the paper which reveals the steps of a typical RNA-seq analysis, highlights the pitfalls and checkpoints that are vital for scientists and biomedical researchers [23], the study which based on machine learning algorithms for the efficient integration of single cell data [19, 24], the identification of known and novel cell types using biological information and the modeling of dynamic changes of cell populations over time [25], the paper which mentions new methods for analyzing scRNA-seq data. In this study observed supervised machine learning algorithms for cell phenotype classification were evaluated using published real and simulated datasets with diverse cell sizes [26]. This paper observes that algorithms and more specifically a quantum clustering technique achieves high accuracy in classifying cells into different cancer types [27]. This study focuses on the application of wet ‘omics technology and dry machine learning approaches together to further develop precision medicine [28].

In our analysis we used two significant books. The first bioinformatics book provides some updates on bioinformatics methods, resources, approaches and genome analysis tools useful for exploring large—volume of biological data [29]. The second book is proper for researchers seeking to process and manage data generated by NGS. It describes algorithms for processing sequencing data and presents useful studies [30].

Based on the findings of the aforementioned studies, our research interest was focused on the formation of an algorithm to generate a classifier based on the expression values of the genes of the original RNA-sequencing dataset, including a dataset containing samples of different types of cancer and showing how supervised classification algorithms can be used to extract significant genes.

It is obvious that this type of data set we chose includes many genes and this has an effect on the results of both methods. The proposed machine learning algorithms developed here can classify well and identify the top most important genes. These classifications were compared with the results of a differential expression analysis. The genes selected by both methods are different. Random genes were selected from the raw samples for the machine learning algorithms and genes with p-value < 0.0001 were selected for the RNA-sequencing analysis. There is remarkable compatibility in the common highly correlated genes between the two methods.

The aim of this research is not to replace differential expression analysis with machine learning algorithms, but to overlap between the two methods in detecting important genes. The original study by [31] for the data set GSE68086 also suggests the use of machine learning algorithms for more accurate analysis. The result in our study shows that while the supervised learning-based gene selection method was used independently of differential expression analysis (i.e., using the genes of all samples and not just the genes with significant differential p-value as input) there is an overlap between the two methods in detecting important genes that play an important role in the development of cancer. The set of selected genes by the proposed method in the GSE68086 data set is shown in Fig. 2. The volcano plot lists the final set of selected genes. The first ten most important genes are protein-coding genes and are the following *KCTD20*, *ZNF185*, *VCL*, *ITGB1*, *F13A1*, *TPP1*, *EIF4G2*, *PRKAR1A*, *CORO1C* and *ABCC3*. The *KCTD20* protein amino acid sequence shows high homology and the expression of this gene may be involved in oncological processes [32]. The *ZNF185* gene expression is involved in the regulation of tumor growth and metastasis [33]. A study shows that the *VCL* gene is significantly associated with cancer [33, 34]. The *ITGB1* gene stimulates cell proliferation, invasion and metastasis [35]. According to studies, the *F13A1* gene is involved in the development of cancer, with dysregulation of excessive platelet activation, thrombosis and its association with inflammation [36]. Also the remaining important genes according to previous studies are cancer-related genes and lead to the development of the tumor [37–40] and a study of *ABCC3* gene whose overexpression indicates poor prognosis in different types of cancer [41]. Our research was limited to the analysis of the first ten significantly expressed genes and the remarkable thing is that the overlap that exists with the algorithms is in the *VCL*, *F13A1* and *ACTN1* genes. The *ACTN1* gene is also a protein-coding gene and associated with cancer [42]. Our results can provide useful information for predicting gene expression. However, we believe that the accuracy of the machine learning method still needs to be improved. As the field of machine learning contains many different supervised classification algorithms, it would be interesting to extend this work by testing the performance of other gene selection algorithms within RNA-sequencing datasets.

In many cases, creating lists of differentially expressed genes is not the final step in the analysis. Further biological knowledge is required by examining changes in gene expression. As it is known in differential expression analysis of RNA-seq data, long or highly expressed genes are more likely to be detected by most existing computational methods. However, such bias against short or lowly expressed genes may distort down-stream data analysis at system biology level. Our study needs to be further improved on this part by developing a computational tool that combines both gene co-expression and RNA-seq data. A gene enrichment analysis performed on the genes identified by the two proposed methods showed that many cancer-related pathways were significantly enriched. However, it would be of interest to extend further biological analysis and interpretation of the results.

## Conclusions

RNA-seq workflow analysis routine was described, focusing on expression quantification and finding differentially expressed genes. Machine learning algorithms are useful tools to improve our determination of gene expression. By comprehensive comparison, we determined that the model based on Random Forest and Gradient Boosting is powerful and robust for differential expression gene's prediction. Taken all together, our study shows that combining the method based on Machine Learning algorithms with RNA-seq analysis significantly improves the recognition of the most important differentially expressed genes and confirms the overlap between these methods.

## Data Availability

The data sets were originally downloaded from the NCBI resource and a full description of the experimental design can be found at https://www.ncbi.nlm.nih.gov/geo/query/acc.cgi?acc=GSE68086. Source code: The R code related to this manuscript can be found on the following link: https://github.com/Calliope-Maria/RNA-seq-analysis.git.

## Abbreviations

BP: Biological process
CC: Cellular components
GEO: Gene expression omnibus
GO: Gene ontology
GTF: Gene transfer format
ID: Identification systems
KEGG: Kyoto encyclopedia of genes and genomes
MF: Molecular function
NGS: Next generation sequencing
PCA: Principal component analysis
RNA-seq: RNA sequencing
TPM: Transcripts-per-million
VST: Variance stabilization transform
